# OLDER ADULTS AND CLIMATE CHANGE ADAPTATION STRATEGIES: A SCOPING REVIEW

**DOI:** 10.1093/geroni/igae098.2322

**Published:** 2024-12-31

**Authors:** Alexandra Holland, Sadaf Milani

**Affiliations:** The University of Texas Medical Branch, Galveston, Texas, United States; The University of Texas Medical Branch, Galveston, Texas, United States

## Abstract

Climate change is one of the biggest threats to human health, resulting in an increase in the frequency and intensity of heatwaves, storms, and other natural disasters. Older adults are at an increased risk from these effects and often have higher rates of morbidity and mortality from climate change-related impacts. As climate change progresses, adaptive strategies are critical for reducing the disproportionate health burden many older adults face. However, little is known about what evidence-based adaptive mechanisms exist that are appropriate for older adults. To address this gap, we conducted a scoping review to identify potential climate change adaptation strategies and solutions for older adults. An Ovid MEDLINE search was done using search terms related to “older adults”, “health”, and “climate change adaptation.” Articles had to be in English and focused on adaptation to climate change for older adults. The initial search returned 399 articles, and after article review, we found 133 articles that met our inclusion criteria. Most of the articles with findings related to climate change adaptation focused on extreme temperatures, such as heatwaves, followed by natural disasters. Emergent themes of the review include older adults’ perception of their own vulnerability and individual adaptive actions and behaviors. There was a lack of research involving systemic adaptive actions aside from national heat plans and built environment modifications. This scoping review can be used to increase older adults’ resilience and inform future systemic adaptive actions to prevent disproportionate morbidity and mortality due to climate change.

